# Determining the Effect of COVID-19 on the Menstrual Cycle Among Women of Reproductive Age Group in the Jazan Region: A Cross-Sectional Study

**DOI:** 10.7759/cureus.32431

**Published:** 2022-12-12

**Authors:** Uma H Chourasia, Ali H Khormi, Hanan A Jawkhab, Shahad I Zoli, Kholoud A Assiri, Shaden A Thurwi, Saleha H Alhazmi, Altaf A Alhazmi, Jawahir M Homadi, Raneem K Zakri, Nada Y Kenani, Ibrahim M Dighriri

**Affiliations:** 1 Department of Obstetrics and Gynaecology, College of Medicine, Jazan University, Jazan, SAU; 2 Faculty of Medicine, Jazan University, Jazan, SAU; 3 Department of Medicine, Jazan University, Jazan, SAU; 4 Department of Medicine and Surgery, Jazan University, Jazan, SAU; 5 Department of Pharmacy, King Abdulaziz Specialist Hospital, Taif, SAU

**Keywords:** coronavirus disease 2019, dysmenorrhea, jazan, figo, covid-19, menstrual cycle

## Abstract

Background: The coronavirus disease 2019 (COVID-19) pandemic has gravely affected the world in various ways. COVID-19 is a major health crisis, with long-term physical and mental health consequences. Many women reported menstrual irregularities during and after the pandemic. The study aimed to assess the effects of COVID-19 on menstrual cycles in females of reproductive age in the Jazan region.

Methodology: A descriptive cross-sectional research design was utilized to conduct the study in Jazan, Saudi Arabia. A structured questionnaire was used to collect data from 346 women aged 18-44 years who had normal menstrual cycles for more than a year before the outbreak and had a history of COVID-19 infection.

Result: The questionnaire was completed by 346 women. Only 144 (41.6%) of the study's respondents were aged 25-34 years. Of the respondents, 283 (81.8%) were university students, and 219 (63.3%) were married. The majority of women (337, 97.4%) were vaccinated against COVID-19. A total of 301 (87.0%) were healthy. Before being infected with COVID-19, 19.70% of the responders had irregular periods, which increased to 59.50% during infection and 33.20% after getting better. There was a relationship between the regularity of menstrual periods during COVID-19 infection and the duration of menstrual periods after COVID-19 (p = 0.035); the frequency of menstrual periods before (p = 0.001), during (p = 0.009), and after (p = 0.001) COVID-19; menstrual period regularity before (p = 0.001) and after (p = 0.001) COVID-19 infection; and pain severity level during (p = 0.001) and after (p = 0.004) COVID-19 infection. Regarding the perception of the impact of COVID-19 on menstrual changes, there was an association between COVID-19 infection and variation in days during two consecutive menstrual cycles (p = 0.001), changes in the duration of menstrual cycles (p = 0.022), delayed or absent menstruation (p = 0.019), and menstruation stopping (p = 0.023).

Conclusion: The research demonstrated the COVID-19 pandemic is an international health problem that affects women, leading to changes in regularity, duration, frequency, and severity of pain. These changes may have a long-term impact on women's reproductive health.

## Introduction

Coronavirus disease 2019 (COVID-19) is a dangerous pandemic illness that has shattered the world with various negative effects. Acute respiratory syndrome coronavirus 2 was first detected in December 2019 in Wuhan, Hubei province [1]. It is considered the seventh human coronavirus. Since then, the virus has spread rapidly over a short period of time to become the most dangerous health problem on a global scale, with varying degrees of burden, severity, psychological consequences, and social effects [2,3]. COVID-19 has had a major effect on people's lives throughout the world, particularly women of reproductive age. The disease itself and control protocols like social distancing and quarantine lead to increased loneliness, anxiety, and depression. This psychological stress during the pandemic has greatly affected female reproductive health [4]. According to a study, the mental state in the United Kingdom has worsened to the end of 2020 compared to the prior COVID-19 outbreak [5].

According to research in the US, the percentage of adult psychological suffering in 2020 was higher than in 2018. In this research, women aged 18-24 years had the most psychological discomfort. Stress may influence women's menstrual cycle [6]. According to the International Federation of Gynecology and Obstetrics (FIGO) systems for nomenclature of symptoms of normal and abnormal uterine bleeding (AUB) in the reproductive years (FIGO AUB system 1), the normal menstrual cycle has a duration of 24-38 days with a normal amount of bleeding up to eight days and regular variation from shortest to longest of < eight days [7,8].

Premenstrual symptoms and dysmenorrhea have been shown to be associated with high-stress levels [9,10]. Popular news reports missing menstrual periods during the pandemic, but there are little data that exist to substantiate these claims [4]. As a result, the purpose of this study is to determine the effect of COVID-19 on the frequency, duration, regularity, and volume of the menstrual cycle in women of reproductive age, and also to define the prevalence of abnormal menstrual cycles due to COVID-19 infection in the Jazan region.

## Materials and methods

Research design and settings

A descriptive cross-sectional research design was utilized to conduct this study in Jazan, Saudi Arabia.

Sample size

Data were collected using snowball and convenience sampling techniques. Raosoft's Sample Size Calculator (Raosoft, Inc., Seattle, WA) was used to calculate the sample size. The minimum needed sample size was 362, based on a 95% confidence interval, a 5% error margin, a 62% expected response, and a total number of women in the Jazan region of 545,721.

Study sample

Women aged 18-44 years who had normal menstrual cycles (according to the FIGO standard) for more than a year prior to the outbreak with a history of COVID-19 infection were recruited in the study.

Tools for data collection

A cross-sectional survey using a modified version of three surveys was used. The first survey included two parts. Part 1 included a structured questionnaire sheet with demographic characteristics such as color, educational level, marital status, nationality, and residence. Part 2 included participants' clinical characteristics, such as medical data and medical histories, such as the presence of chronic disease and the status of COVID-19 vaccination. The second survey contained the assessment sheet for the effect of COVID-19 on the duration, regularity, pain severity, frequency of menstrual periods, and amount of blood loss; each question was answered with either "yes" or "no." The third survey was conducted to learn about women's perceptions of the effect of COVID-19 on menstrual changes. It was classified according to the level of agreement (strongly disagree, disagree, neutral, agree, and strongly agree).

Pilot study

A pilot study was carried out to evaluate the viability, objectivity, clarity, and application of study instruments and was conducted on 10% of the study sample. The necessary alterations were made and were removed from the study sample.

Data collection process

Three surveys were prepared with the structured questionnaire after reviewing the related literature. The access link was prepared and distributed to participants who gave their consent via online platforms. The link had also been distributed directly to women. The tools were tested for reliability using Cronbach's alpha coefficient.

Statistical design

After the data were gathered, they were reviewed, coded, and entered into a computer for analysis. The Statistical Package for the Social Sciences (SPSS) for Windows (IBM Corp., Armonk, NY) was used for data analysis. All continuous variables were converted to categorical variables. Frequency and percentage were used to express categorical data. The chi-square test was utilized to compare variables using categorical data. The probability was set at 0.05.

Ethical consideration

The proposal was accepted by Jazan University's Standing Committee for Scientific Research with the number REC-44/03/323. Online informed consent was collected in Arabic. Subjects were told that their involvement was entirely voluntary and that the information would be processed secretly and anonymously.

## Results

The study included 346 participants between the ages of 18 and 44 years who had previously been infected with COVID-19. Only 144 respondents (41.6%) in the study were between the ages of 25 and 34 years. Of the respondents, 291 (84.1%) were white. The majority of respondents (283, 81.8%) were university students, 219 (63.3%) were married, and 118 (34.1%) were employed. The majority of participants (97.4%) were of Saudi nationality. A total of 218 (63.0%) were living in cities. Almost all of the participants (333, 96.2%) were non-smokers, 337 (97.4%) were vaccinated against COVID-19, and 181 (52.3%) were vaccinated against influenza. A total of 247 (71.4%) participants received the COVID-19 vaccine before being infected. Of the respondents, 301 (87.0%) were healthy and only 45 (13.0%) had chronic diseases (Table 1).

**Table 1:** Demographic characteristics of 346 females

Demographic characteristics		Number (N)	Percentage (%)
Age	18-24	118	34.1
	25-34	144	41.6
	35-44	84	24.3
Color	White	291	84.1
	Other	55	15.9
Education level	Secondary or less	40	11.6
	University	283	81.8
	Postgraduate	23	6.6
Marital status	Married	219	63.3
	Single	113	32.7
	Widow	2	0.6
	Divorced	12	3.5
Employment status	Employed	118	34.1
	Not employed	228	65.9
Nationality	Saudi	337	97.4
	Non-Saudi	9	2.6
Residence	Village	128	37.0
	City	218	63.0
Smoking status	Non-smoker	333	96.2
	Smoker	13	3.8
COVID-19 vaccine status	Vaccinated	337	97.4
	Not vaccinated	9	2.6
Time of receipt of the COVID-19 vaccine	Before being affected	247	71.4
	After being affected	99	28.6
Influenza vaccine status	Vaccinated	181	52.3
	Not vaccinated	165	47.7
Any type of chronic disease	Yes	45	13.0
	No	301	87.0

**Figure 1:**
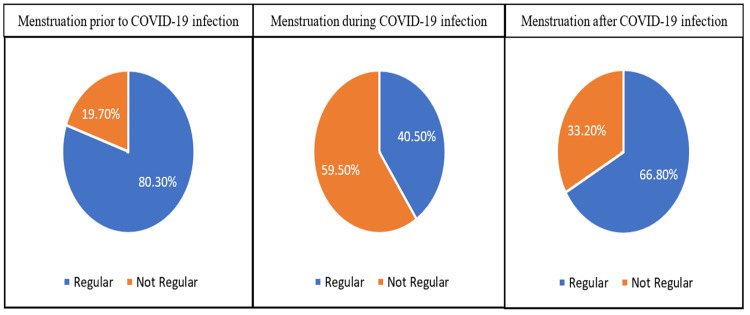
Regularity and irregularity of menstruation prior to, during, and after COVID-19 infection

According to prevalence, 19.70% of respondents reported having irregular menstrual cycles before COVID-19, 59.50% during infection by COVID-19, and 33.20% after COVID-19 infection (Figure 1).

Our findings reveal that the regularity of menstrual periods during COVID-19 infection is not related to the number of pregnancies (p = 0.361) or abortions (p = 0.734). There is a relationship between the regularity of menstrual periods during COVID-19 infection and the duration of menstrual periods after COVID-19 (p = 0.035); frequency of menstrual periods before (p = 0.001), during (p = 0.009), and after (p = 0.001) COVID-19; regularity of menstrual period before (p = 0.001) and after (p = 0.001) COVID-19 infection; and pain severity level during (p = 0.001) and after (p = 0.004) COVID-19 infection (Table 2).

**Table 2:** Effects of COVID-19 on the menstrual period of the respondents

Variable		Regularity of menstrual periods during the COVID-19 infection		Total, N (%)	P-value
		Regular, N (%)	Not regular, N (%)		
Number of pregnancies	None	61 (17.6%)	92 (26.6%)	153 (44.2%)	0.361
	Three times or less	51 (14.7%)	62 (17.9%)	113 (32.7%)	
	Three times or more	28 (8.1%)	52 (15.0%)	80 (23.1%)	
Number of abortions	None	106 (30.6%)	160 (46.2%)	266 (76.9%)	0.734
	One time	22 (6.4%)	25 (7.2%)	47 (13.6%)	
	Twice	7 (2.0%)	14 (4.0%)	21 (6.1%)	
	Three times or more	5 (1.4%)	7 (2.0%)	12 (3.5%)	
Duration of the menstrual period before COVID-19	1-3 days	10 (2.9%)	16 (4.6%)	26 (7.5%)	0.546
	4-6 days	91 (26.3%)	122 (35.3%)	213 (61.6%)	
	More than 6 days	39 (11.3%)	68 (19.7%)	107 (30.9%)	
Duration of the menstrual period during COVID-19	1-3 days	15 (4.3%)	11 (3.2%)	26 (7.5%)	0.113
	4-6 days	93 (26.9%)	154 (44.5%)	247 (71.4%)	
	More than 6 days	32 (9.2%)	41 (11.8%)	73 (21.1%)	
Duration of the menstrual period after COVID-19	1-3 days	18 (5.2%)	18 (5.2%)	36 (10.4%)	0.035
	4-6 days	84 (24.3%)	105 (30.3%)	189 (54.6%)	
	More than 6 days	38 (11.0%)	83 (24.0%)	121 (35.0%)	
Frequency of menstrual period (between two menses) before COVID-19	Less than 24 days	36 (10.4%)	43 (12.4%)	79 (22.8%)	0.001
	Between 24 and 38 days	91 (26.3%)	107 (30.9%)	198 (57.2%)	
	More than 38 days	13 (3.8%)	56 (16.2%)	69 (19.9%)	
Frequency of menstrual period (between two menses) during COVID-19	Less than 24 days	39 (11.3%)	30 (8.7%)	69 (19.9%)	0.009
	Between 24 and 38 days	87 (25.1%)	148 (42.8%)	235 (67.9%)	
	More than 38 days	14 (4.0%)	28 (8.1%)	42 (12.1%)	
Frequency of menstrual period (between two menses) after COVID-19	Less than 24 days	44 (12.7%)	60 (17.3%)	104 (30.1%)	0.001
	Between 24 and38 days	79 (22.8%)	84 (24.3%)	163 (47.1%)	
	More than 38 days	17 (4.9%)	62 (17.9%)	79 (22.8%)	
Regularity of menstrual period before COVID-19 infection	Regular	134 (38.7%)	144 (41.6%)	278 (80.3%)	0.001
	Not regular	6 (1.7%)	62 (17.9%)	68 (19.7%)	
Regularity of menstrual period after COVID-19	Regular	127 (36.7%)	104 (30.1%)	231 (66.8%)	0.001
	Not regular	13 (3.8%)	102 (29.5%)	115 (33.2%)	
Pain severity level associated with menstrual period before COVID-19 infection	Mild	50 (14.5%)	60 (17.3%)	110 (31.8%)	0.347
	Moderate	73 (21.1%)	113 (32.7%)	186 (53.8%)	
	Severe	17 (4.9%)	33 (9.5%)	50 (14.5%)	
Pain severity level associated with menstrual period during COVID-19 infection	Mild	42 (12.1%)	12 (3.5%)	54 (15.6%)	0.001
	Moderate	77 (22.3%)	169 (48.8%)	246 (71.1%)	
	Severe	21 (6.1%)	25 (7.2%)	46 (13.3%)	
Pain severity level associated with menstrual period after COVID-19	Mild	44 (12.7%)	34 (9.8%)	78 (22.5%)	0.004
	Moderate	68 (19.7%)	117 (33.8%)	185 (53.5%)	
	Severe	28 (8.1%)	55 (15.9%)	83 (24.0%)	

After COVID-19 infection, 38 (11.0%) vs. 83 (24.0%) women with regular and irregular menstrual periods, respectively, had menstrual periods that lasted more than six days; 17 (4.9%) vs. 62 (17.9%) women with regular and irregular menstrual periods, respectively, had a frequency of menstrual periods that lasted more than 38 days; and 28 (8.1%) vs. 55 (15.9%) women with regular and irregular menstrual periods, respectively, had severe pain during their menstrual period (Table 2).

Only 107 (30.9%) women had a menstrual cycle that lasted more than six days before the COVID-19 infection, compared to 73 (21.1%) during the COVID-19 infection, with a significant increase of 121 (35.0%) after COVID-19 infection. Regarding the frequency of menstrual cycles, it can be observed that around 69 women (19.9%) had the frequency of two menses more than 38 days before COVID-19, compared to 42 women (12.1%) during and 79 women (22.8%) after COVID-19. Regarding the regularity of menstrual periods, 278 (80.3%) women reported having a regular menstrual cycle before COVID-19, compared to 231 (66.8%) after COVID-19. A total of 186 (53.8%) women experienced moderate pain levels before COVID-19, and this percentage increased statistically to 246 (71.1%) during COVID-19. However, after COVID-19, the percentage returned to 185 (53.5%) (Table 2).

Only 127 (37.4%) women reported that they had bleeding between periods before COVID-19, compared to 75 (21.7%) during COVID-19 and 39 (11.3%) after COVID-19. In terms of missing the menstrual cycle, 83 (24.0%) women answered yes they missed it before COVID-19, 72 (20.8%) during, and 54 (15.6%) after COVID-19 (Table 3).

**Table 3:** The impact of COVID-19 on the occurrence of menstrual cycle bleeding and missing

Item		Regularity of menstrual periods during the COVID-19 infection		Total, N (%)	P-value
		Regular, N (%)	Not regular, N (%)		
Before your infection with COVID-19, did you experience bleeding between periods?	Yes	86 (25.3%)	41 (12.1%)	127 (37.4%)	0.001
	No	34 (10.0%)	136 (40.0%)	170 (50.0%)	
	I don't know	15 (4.4%)	28 (8.2%)	43 (12.6%)	
During your infection with COVID-19, did you experience bleeding between periods?	Yes	30 (8.7%)	45 (13.0%)	75 (21.7%)	0.927
	No	110 (31.8%)	161 (46.5%)	271 (78.3%)	
After your infection with COVID-19, did you experience bleeding between periods?	Yes	16 (4.6%)	23 (6.6%)	39 (11.3%)	0.939
	No	124 (35.8%)	183 (52.9%)	307 (88.7%)	
Before your infection with COVID-19, did you miss your period?	Yes	28 (8.1%)	55 (15.9%)	83 (24.0%)	0.152
	No	112 (32.4%)	151 (43.6%)	263 (76.0%)	
During your infection with COVID-19, did you miss your period?	Yes	19 (5.5%)	53 (15.3%)	72 (20.8%)	0.006
	No	121 (35.0%)	153 (44.2%)	274 (79.2%)	
After your infection with COVID-19, did you miss your period?	Yes	20 (5.8%)	34 (9.8%)	54 (15.6%)	0.577
	No	120 (34.7%)	172 (49.7%)	292 (84.4%)	

Regarding the perception of the impact of COVID-19 on menstrual changes, there was a relationship between the regularity of menstrual periods during the COVID-19 infection and changes in the number of days between two menstrual cycles (p = 0.001), changes in the length of menstrual cycles (p = 0.022), delayed or absent menstruation (p = 0.019), and menstruation stopping (p = 0.023) (Table 4).

**Table 4:** Perception of the effects of the COVID-19 infection on menstrual changes

Item		Regularity of menstrual periods during the COVID-19 infection		Total, N (%)	P-value
		Regular, N (%)	Not regular, N (%)		
Does COVID-19 cause changes in the number of days between two consecutive menstrual cycles?	Strongly disagree	8 (2.3%)	11 (3.2%)	19 (5.5%)	0.001
	Disagree	33 (9.5%)	18 (5.2%)	51 (14.7%)	
	Neutral	36 (10.4%)	47 (13.6%)	83 (24.0%)	
	Agree	42 (12.1%)	68 (19.7%)	110 (31.8%)	
	Strongly agree	21 (6.1%)	62 (17.9%)	83 (24.0%)	
Does COVID-19 infection cause changes in the length of menstrual cycles?	Strongly disagree	11 (3.2%)	9 (2.6%)	20 (5.8%)	0.022
	Disagree	33 (9.5%)	25 (7.2%)	58 (16.8%)	
	Neutral	36 (10.4%)	56 (16.2%)	92 (26.6%)	
	Agree	38 (11.0%)	70 (20.2%)	108 (31.2%)	
	Strongly agree	22 (6.4%)	46 (13.3%)	68 (19.7%)	
Does COVID-19 infection cause changes in the amount of blood loss that occurred during the cycle?	Strongly disagree	10 (2.9%)	9 (2.6%)	19 (5.5%)	0.072
	Disagree	26 (7.5%)	22 (6.4%)	48 (13.9%)	
	Neutral	45 (13.0%)	60 (17.3%)	105 (30.3%)	
	Agree	31 (9.0%)	66 (19.1%)	97 (28.0%)	
	Strongly agree	28 (8.1%)	49 (14.2%)	77 (22.3%)	
Does COVID-19 infection cause bleeding between periods (regardless of the amount)?	Strongly disagree	14 (4.0%)	14 (4.0%)	28 (8.1%)	0.344
	Disagree	41 (11.8%)	46 (13.3%)	87 (25.1%)	
	Neutral	39 (11.3%)	71 (20.5%)	110 (31.8%)	
	Agree	29 (8.4%)	43 (12.4%)	72 (20.8%)	
	Strongly agree	17 (4.9%)	32 (9.2%)	49 (14.2%)	
Does COVID-19 infection cause some periods to be delayed or absent?	Strongly disagree	12 (3.5%)	10 (2.9%)	22 (6.4%)	0.019
	Disagree	36 (10.4%)	30 (8.7%)	66 (19.1%)	
	Neutral	37 (10.7%)	53 (15.3%)	90 (26.0%)	
	Agree	32 (9.2%)	71 (20.5%)	103 (29.8%)	
	Strongly agree	23 (6.6%)	42 (12.1%)	65 (18.8%)	
Does COVID-19 infection cause menstruation to stop?	Strongly disagree	15 (4.3%)	16 (4.6%)	31 (9.0%)	0.023
	Disagree	51 (14.7%)	47 (13.6%)	98 (28.3%)	
	Neutral	35 (10.1%)	57 (16.5%)	92 (26.6%)	
	Agree	25 (7.2%)	60 (17.3%)	85 (24.6%)	
	Strongly agree	14 (4.0%)	26 (7.5%)	40 (11.6%)	
Does COVID-19 infection cause changes in menstrual pain that occur just before or during menstruation?	Strongly disagree	7 (2.0%)	8 (2.3%)	15 (4.3%)	0.034
	Disagree	32 (9.2%)	23 (6.6%)	55 (15.9%)	
	Neutral	35 (10.1%)	51 (14.7%)	86 (24.9%)	
	Agree	42 (12.1%)	73 (21.1%)	115 (33.2%)	
	Strongly agree	24 (6.9%)	51 (14.7%)	75 (21.7%)	

Of the respondents, 83 (24.0%) strongly agreed that COVID-19 caused changes in the number of days between two menstrual cycles, while 77 (22.3%) women strongly agreed that COVID-19 infection caused changes in the amount of blood lost during the menses. Furthermore, 49 (14.2%) women strongly agreed when asked whether COVID-19 caused bleeding between periods. A total of 65 (18.8%) study participants strongly agreed that COVID-19 caused a delay or absence in the menstrual cycle (Table 4).

## Discussion

COVID-19 is a dangerous pandemic disease that has a detrimental impact on everyone in the world. During the COVID-19 pandemic, women faced stressful times that have been shown to impact their menstrual cycles [4]. Therefore, the objective of the current study was to evaluate the effects of the COVID-19 epidemic on women's reproductive health and menstrual cycle. Our findings revealed that the menstrual cycles changed during and after COVID-19, in terms of the number of days between successive cycles, the quantity of blood, or menses length. This may be attributed to the psychological disorders that influence these irregularities in the menstrual cycle, particularly the severe stress and sadness resulting from COVID-19. In terms of duration or length, the current study found that the duration of the menstrual cycle changes during COVID-19 infection compared to before COVID-19. This result is in line with a previous study, which reported that among women in their study, the cycle's median duration had not changed, but that its range had grown noticeably larger and the minimum and highest recorded cycle lengths had shrunk [4]. This study has shown that the COVID-19 pandemic has had a significant negative impact on the reproductive health of the female population aged 18 to 44 years. This is in line with the previous study [4].

According to prevalence, 19.70% of the respondents reported abnormal menstrual cycles before COVID-19, 59.50% during infection by COVID-19, and 33.20% after healing from COVID-19. This result is in agreement with a study conducted in Turkey, which reported that more than one-fourth of women experienced irregular menstrual cycles during COVID-19 [11]. COVID-19 infection leads to irregular menstrual cycles. Previous research reported that the incidence of menstrual regulation was more than one-tenth lower during the pandemic [12]. Regarding the severity of pain associated with menses, the current result revealed half of the women experienced moderate pain levels before COVID-19, and this percentage increased statistically during COVID-19 and decreased after COVID-19. This result is in harmony with the report that the studied sample had experienced more painful periods during COVID-19 [4].

The current study found that during and after the COVID-19 infection, women had less bleeding between periods than they did before the infection. According to research performed in Iraq and Jordan, 18.8% of women had bleeding between periods during COVID-19 [13]. Regarding missed periods, the current study showed that fewer women had experienced missed periods during and after COVID-19 compared to before COVID-19. This result contrasts with a previous study that reported that the number of missed periods increased significantly as a result of COVID-19 [4].

The current study identified a link between the increase in the prevalence of menstrual cycle irregularity in women and the COVID-19 pandemic. To protect a person's reproductive health, these results show how important it is to take steps to improve their physical and mental health during an outbreak.

Limitations

First, the design of the study was cross-sectional; therefore, it cannot demonstrate a causal link. Second, because the study was limited to the Jazan region, the findings cannot be generalized to the whole country. Third, because the study was conducted after women were infected with COVID-19 and we asked them some questions before they were infected, some women may not recall some information. Finally, the study did not investigate the effects of stress and anxiety on menstruation.

Recommendations

Insufficient quality study on COVID-19 and menstrual, despite the fact that new studies on the topic are constantly being published, is a reflection of the general direction of research not concerned with women's health, particularly pregnancy, so there is a need to conduct further research in women’s reproductive health. Future research should include ongoing multimodal evaluation at various intervals throughout the pandemic, including measurements of body mass index, hormone levels, and ovulation, as well as validated ongoing assessments of mental health. Continuous educational programs for women on how to deal with this new virus should teach women how to maintain proper adherence to preventive measures to avoid any further infection. There is a need to conduct online emotional support sessions to reassure affected women and provide them the chance to obtain medical advice during their illness and isolation period.

## Conclusions

The study showed that the COVID-19 pandemic is considered one of the most serious health crises and has a significant impact on women’s health, leading to many negative changes for all people, especially women of reproductive age. These changes can negatively affect the menstrual cycle, leading to an increased prevalence of irregularity, increased severity of pain, increased length, a change in the number of days between successive cycles, and a change in the quantity of blood loss. All of these changes can have serious long-term effects on reproductive health. Therefore, we need to look for solutions to menstrual cycle irregularity due to COVID-19 infection, and we need prospective research to validate these results and assess the duration of these menstrual abnormalities.
